# Open surgical resection of giant common hepatic artery aneurysm after failed endovascular repair

**DOI:** 10.1016/j.jvscit.2026.102183

**Published:** 2026-02-12

**Authors:** Javier Fernandez, Jorge Rey, Alan Livingstone

**Affiliations:** a Department of Medicine, University of Miami Leonard M. Miller School of Medicine, Miami, FL; b DeWitt Daughtry Family Department of Surgery, Division of Vascular Surgery, Leonard M. Miller School of Medicine, University of Miami, Miami, FL; c DeWitt Daughtry Family Department of Surgery, Division of Surgical Oncology, Leonard M. Miller School of Medicine, University of Miami, Miami, FL

**Keywords:** Hepatic artery aneurysm, Visceral artery aneurysm, Giant aneurysm, Open surgical repair, Endovascular repair

## Abstract

Hepatic artery aneurysms represent 20% of visceral artery aneurysms and carry a high risk of fatal outcomes. We describe an 8.6-cm common hepatic artery aneurysm previously treated with endovascular stenting and coiling that progressively enlarged and was complicated by endoleak and mural thrombus. Open partial resection was performed without reconstruction, and the patient made a full recovery with no recurrence at the 2-year follow-up. This case underscores the therapeutic challenges in treating giant aneurysms and highlights the importance of assessing collateral circulation when considering an aneurysmectomy.

Hepatic artery aneurysms (HAAs) represent the second most common type of visceral artery aneurysm, accounting for approximately 20% of all visceral aneurysms after splenic artery aneurysms.^1^ Despite their rarity, HAAs carry significant clinical importance owing to their substantial risk of rupture,^2^ with mortality rates exceeding 30% in ruptured cases.^2^, ^3^, ^4^, ^5^

Most true HAAs result from atherosclerosis or mediointimal degeneration and most commonly involve the common hepatic artery (CHA). HAAs are typically asymptomatic and discovered incidentally on imaging,^6^ although patients may present with life-threatening complications, including hemobilia or intraperitoneal hemorrhage.^2^^,^^7^ Current guidelines recommend repair of asymptomatic HAAs surpassing 3 cm,^8^^,^^9^ with endovascular intervention representing the preferred first-line approach when anatomically feasible.

We present a case of a giant CHA aneurysm measuring 8.6 cm that progressively enlarged despite prior endovascular repair. Owing to continued aneurysmal growth and failure of prior interventions, open surgical resection was performed, highlighting the diagnostic and management challenges of these rare vascular lesions. The patient provided written informed consent for publication of the case details and associated images in accordance with journal guidelines and the Declaration of Helsinki.

## Case Report

**Fig 1:**
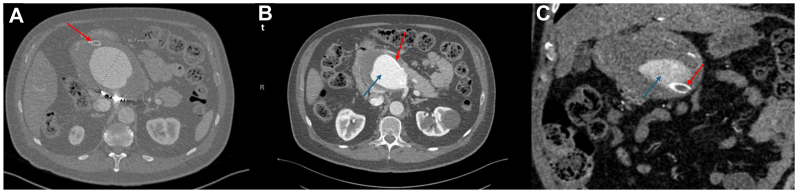
**(A)** Axial computed tomography (CT) image demonstrating a fusiform aneurysm arising from the common hepatic artery (CHA) with a maximal diameter of 8.6 cm (*red arrowhead* indicates the distal portion of an occluded endovascular stent). **(B)** Axial view showing a free-floating stent coursing along the anterior aspect of the aneurysm, with a contrast-enhanced region consistent with an endoleak (*red arrowhead* indicates the stent; *blue arrowhead* indicates the endoleak). **(C)** Coronal image depicting the proximal portion of the stent near the takeoff of the CHA, along with the associated endoleak (*red arrowhead* indicates the stent; *blue arrowhead* indicates the endoleak).

A Hispanic man in his 60s with a history of hypertension and former tobacco use was initially diagnosed with a large CHA aneurysm and underwent endovascular repair with stent placement and coil embolization. Seven years later, incidental computed tomography (CT) imaging revealed a recurrent aneurysm measuring 9.8 × 7.0 cm with a free-floating stent, which was not pursued or intervened on at the time at an outside institution. Subsequent evaluation with CT angiography demonstrated a markedly enlarged fusiform aneurysm with a maximal diameter of 8.6 cm (Fig 1, *A*) and a longitudinal extent of 14.3 cm. The previously placed stent was occluded and displaced, with extensive surrounding mural hematoma and an endoleak at the proximal stent margin (Fig 1, *B* and *C*). The hepatic arteries received collateral blood flow from the pancreaticoduodenal arcade off the superior mesenteric artery.

## Treatment

**Fig 2:**
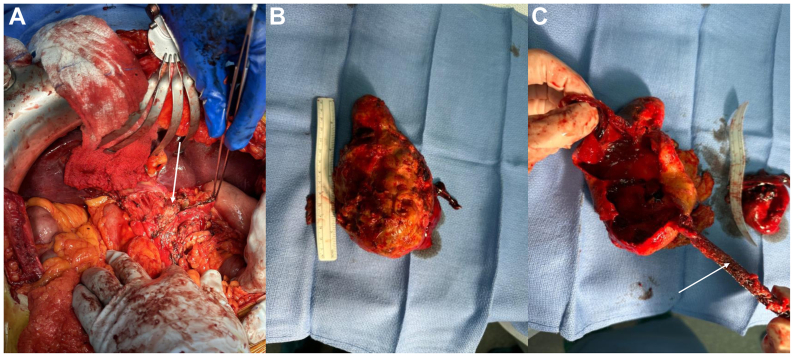
**(A)** Intraoperative exposure of the common hepatic artery (CHA) aneurysm (indicated by the *white arrowhead*). **(B)** Resected aneurysm measuring 15.0 cm in length. **(C)** Dissected hepatic artery aneurysm (HAA) with mural thrombus and free floating stent (*white arrowhead*).

The procedure was performed via a right subcostal incision to access the peritoneal cavity. The stomach and omental attachments were mobilized through the lesser omentum based on preoperative imaging, which demonstrated hepatic perfusion primarily via collaterals from the superior mesenteric artery coursing posterior to the aneurysm. The gastrocolic ligament was divided to enter the lesser sac and several omental adhesions surrounding the aneurysm were dissected carefully. The CHA was then fully exposed (Fig 2, *A*). Given aneurysmal extension into the hepatic hilum, the liver transplant surgery team provided assistance with vascular control.

Intraoperative ultrasound examination confirmed adequate hepatic perfusion, and a vessel loop was placed around the CHA. After obtaining proximal control and placing a vessel loop around the portal triad, the CHA and splenic artery were clamped at the celiac axis and repeat ultrasound examination confirmed continued collateral flow. The distal CHA was ligated and the aneurysmal sac opened without retrograde bleeding. Partial aneurysm resection was performed, with approximately 75% of the sac excised. The posterior wall, which was densely adherent to the pancreas and surrounding structures owing to inflammation, was left in situ to avoid injury to these vital structures. The proximal and distal ends were ligated given preserved collateral flow. The resected specimen revealed organized thrombus, a displaced stent, and coiling material (Fig 2, *B*), measuring 15 cm in length (Fig 2, *C*). Intraoperative ultrasound examination confirmed preserved flow through the collaterals into both hepatic arteries along with the splenic and left gastric artery.

In the event of inadequate hepatic perfusion after testing clamping, the ipsilateral thigh was prepared for saphenous vein harvest to allow arterial reconstruction from the supraceliac aorta, infrarenal aorta, or right iliac artery depending on anatomic suitability.^10^^,^^11^

## Outcome and follow-up

The patient's postoperative course was complicated by necrotizing pancreatitis and pancreatic fistula formation that was managed nonoperatively and through interventional radiology-guided drainage and antibiotics. The patient ultimately made a full recovery, with normal hepatic function and no evidence of aneurysmal recurrence at 2 years postoperatively. He remains on long-term surveillance with annual CT imaging to monitor for recurrent aneurysm formation or collateral-related complications. Given the absence of residual lumen and preserved collateral hepatic perfusion, no interval intervention is currently planned; however, imaging frequency may be extended after 3 to 5 years of stability.

## Discussion

Giant visceral aneurysms are defined as those ≥5 cm in diameter.^12^ Our case at 8.6 cm diameter represents a particularly large example with progressive enlargement despite prior endovascular treatment. Among giant HAAs, the majority present with abdominal pain, although smaller HAAs are typically asymptomatic and discovered incidentally, as seen in our patient.^13^^,^^14^ HAAs carry the highest rupture risk among splanchnic aneurysms, emphasizing the importance of early detection.^6^ Hypertension and tobacco use, both present in our patient, are common risk factors that promote endothelial injury and vessel wall degeneration.^15^^,^^16^

The Society for Vascular Surgery advises repair for HAAs ≥3 cm or those that are symptomatic or rapidly expanding.^8^ Endovascular techniques are increasingly preferred owing to lower morbidity, although reintervention rates are notably higher.^3^ In our case, preoperative imaging revealing adequate collateral flow allowed for safe partial aneurysmectomy. Intraoperative ultrasound examination verified continuous hepatic perfusion throughout the procedure, preventing ischemic complications such acute liver failure.^17^^,^^18^

Alternative surgical approaches were considered during operative planning, including ligation, resection with arterial reconstruction, and aneurysmorrhaphy. Endoaneurysmorrhaphy represents a well-established technique for HAA repair, wherein the aneurysm sac is opened after proximal and distal control, evacuated, and the arterial wall is plicated or reconstructed without complete excision of the aneurysmal tissue.^19^^,^^20^ This tissue-sparing approach offers several theoretical advantages, including a lower risk of injury to adjacent structures such as the portal vein and common bile duct through more limited dissection compared with complete aneurysmal resection.

We ultimately elected to proceed with partial resection and ligation based on several considerations. The extension of the aneurysm into the hepatic hilum created uncertainty regarding our ability to obtain control of all inflow and outflows vessels, particularly given the complex anatomy at the hepatic bifurcation. Additionally, the presence of organized thrombus and failed endovascular hardware within the sac raised concern for potential infectious or inflammatory complications if residual aneurysmal wall were left in situ. Given the documented collateral hepatic perfusion, we determined that arterial reconstruction would not be necessary, allowing for simple ligation after partial resection. In retrospect, endoaneurysmorrhaphy may have been a reasonable alternative approach that may have decreased the extent of dissection around the pancreaticoduodenal arcade and potentially decreased the risk of postoperative pancreatitis. The extensive dissection required for our partial resection approach may have contributed to this complication through mechanical manipulation of the arcade and transient ischemia to pancreatic tissue.

## Conclusions

Overall, this case highlights the technical and perioperative challenges of managing large, complex HAAs after failed endovascular repair. Careful preoperative assessment of collateral hepatic circulation CT angiography is critical, because the presence of adequate extrahepatic collaterals permits safe hepatic artery ligation or resection without the need for arterial reconstruction. Multidisciplinary collaboration is essential to optimize treatment selection, with open surgical repair remaining an important option for cases with unfavorable anatomy or endovascular treatment failure.

## Funding

None.

## Disclosures

None.
